# Characterizing Measurement-Based Care in the Texas Youth Depression and Suicide Research Network (TX-YDSRN)

**DOI:** 10.1007/s10578-023-01653-3

**Published:** 2024-02-10

**Authors:** Holli Slater, Yasmin AlZubi, Afsaneh Rezaeizadeh, Jennifer L. Hughes, April Gorman, Taryn L. Mayes, Joshua S. Elmore, Eric A. Storch, Sarah M. Wakefield, Madhukar H. Trivedi

**Affiliations:** 1 Center for Depression Research and Clinical Care, Peter O’Donnell Jr. Brain Institute and Department of Psychiatry, University of Texas Southwestern Medical Center, 5323 Harry Hines Blvd, Dallas, TX 75390-9119 USA; 2 Nationwide Children’s Hospital, The Ohio State University, Columbus, OH USA; 3 Department of Psychiatry & Behavioral Sciences, Baylor College of Medicine, Houston, TX USA; 4 Department of Psychiatry, Texas Tech University Health Sciences Center, Lubbock, TX USA

**Keywords:** Measurement-based care, Depression, Children, Assessment, Adolescents

## Abstract

Integration of measurement-based care (MBC) into clinical practice has shown promise in improving treatment outcomes for depression. Yet, without a gold standard measure of MBC, assessing fidelity to the MBC model across various clinical settings is difficult. A central goal of the Texas Youth Depression and Suicide Research Network (TX-YDSRN) was to characterize MBC across the state of Texas through the development of a standardized tool to assess the use of MBC strategies when assessing depression, anxiety, side effects, and treatment adherence. A chart review of clinical visits indicated standardized depression measures (71.2%) and anxiety measures (64%) were being utilized across sites. The use of standardized measures to assess medication adherence and side effects was limited to less than six percent for both, with the majority utilizing clinical interviews to assess adherence and side effects; yet medication was changed in nearly half. Rates of utilization of standardized measures for participants with multiple MBC forms were similar to those who only provided one form.

## Introduction

Depression is a common condition characterized by persistent feelings of sadness and hopelessness, as well as an array of other symptoms [3]. Feelings of depression and thoughts of suicide are increasing among children and adolescents; 42.3% of youth aged 12–17 reported in the 2021 Youth Risk Behavior Survey that they had felt so sad or hopeless in the past 12 months that they stopped doing regular activities [9]. The COVID pandemic has been associated with increased rates of depression among youth, particularly amongst females [32].

Depression in youth is treatable, with both pharmacotherapy and psychotherapy demonstrating benefit [52]. Yet, accurate identification of cases is a critical first step. In 2016, the U.S. Preventive Services Task Force recommended screening for major depressive disorder (MDD) for adolescents aged 12–18 years of age [50]. Currently, depression treatment for adolescents includes psychopharmacology (the most common form of treatment), psychotherapy, or a combination of both, all with the primary goal of achieving full remission of symptomology [14, 45]. All three treatment options are supported by empirical evidence, though establishing stable remission of depressive symptoms remains a challenge, and relapse often occurs [14]. Despite some evidence of improved outcomes through the adoption of universal screening in adults [25], the consistent use of measurement-based care and the utilization of collaborative care models in pediatric settings are limited [35].

### Major Depressive Disorder and Current Treatment Guidelines

Depression is a chronic and recurrent illness which affects all genders and can occur at any age [12]. Often, the onset of initial symptoms of depression are present before the age of 17 and can proceed into adulthood [40], making early detection essential [2, 4]. The likelihood of being affected by a recurrent episode is increased with every subsequent episode [27]. Furthermore, successful treatment in youth can decrease the likelihood of life altering factors that may impact the transition into adulthood, such as substance use behaviors and suicide risk [34].

Primary care settings should be equipped to not only identify, but also to either initiate or connect youth to evidence-based practices [19, 52]. With MDD, the treatment goal is to achieve full remission of symptoms, prevent relapse, and return patients to the level of premorbid functioning [28, 38, 45]. Screening for adolescent depression is recommended based on multiple guidelines, and often uses established measures [19]. Use of measures across treatment is less standard, and effective use involves answering questions regarding how often to get measures, how to build assessment into clinic systems for consistency and efficiency, and how to use measures effectively to aid in clinical decision-making [35].

With the pervasive occurrence of MDD, implementing effective treatment is essential to the safety and well-being of those impacted by depression. However, the current treatment for depression does not fully align with the “best practice” standards, resulting in a disorganized and irregular approach [22]. Depression treatment ineffectiveness is indicated by reports of side effects, residual symptoms, and impaired functioning among patients receiving treatment [45]. Studies have demonstrated that when youth in primary care settings are screened and offered high quality depression treatments, this results in improved clinical outcomes [5, 6, 37, 55].

In the absence of objective data to review, practitioners engage in a trial-and-error approach to medication adjustments impacting how outcomes are assessed [45]. Treatment effectiveness is measured by the real-time subjective assessment of the patient rather than a review of longitudinal symptom severity [33]. This method of treatment evaluation is more reflective of the impacts of recent stressors rather than overall treatment response.

When remission is not achieved by antidepressants (with or without psychotherapy), a variety of strategies are available to clinicians, including switching to another medication from the same class, transitioning to an antidepressant from another class, augmenting the antidepressant medication with another medication, or adding psychotherapy [23]. Since the variation in treatment options is so wide, a trial-and-error approach may prolong inadequate treatment, burden the patient due to frequent changes, result in the development of side effects, or result in changing a medication that could have been effective if titrated [10, 23].

### Measurement-Based Care and Depression

Measurement-based care (MBC) in psychiatry is defined as “the routine measurement of symptoms and side effects using validated clinical measurement instruments at each treatment visit to objectify the assessment, tolerability, functioning, and quality of life in patients with a psychiatric disorder” [1]. MBC is used in the treatment of other diseases (e.g., blood sugar for diabetes, blood pressure and cholesterol for cardiac disease), yet its use is not standard practice in psychiatric care [31, 33].

Although MBC is not standard in psychiatry, when implemented in the clinical care setting, MBC has improved outcomes in both adult and adolescent patients with depression [1, 38, 39]. MBC in the primary care setting yields improved efficiency and accuracy of assessment relative to standard practice, as well as increased treatment adherence [2, 39]. The use of MBC to monitor the progress of patients may increase rigor of mental health treatment and increase the likelihood of full remission [31].

When systematic MBC procedures are applied, adults treated in primary care clinics for depression have similar outcomes to patients treated in psychiatric settings; this may be facilitated by the use of MBC to aid in providing precision and consistency in assessment, monitoring, and treatment of the disease [17, 22, 48]. The MBC approach includes two main components: 1) routine data collection with standardized and validated measures and 2) use of this data to guide treatment [35]. More specifically, routine data collection should include a standardized assessment of symptoms, side effects, and treatment adherence, and guided treatment should include point-of-care decision-making for treatment and consistent follow-up visits. When compared with standard of care, the use of MBC is linked to rates of remission twice as high [18] and has currently been implemented in depression treatment guidelines [20, 25]. As clinicians rarely administer serial measurements in their practice, the MBC approach relies on patient self-report assessments for both screening and management of depression.

Patient education and involvement in managing their illness can also improve outcomes [29]. An additional benefit for the utilization of MBC guidelines includes strengthening doctor-patient relationships, thus encouraging open and accurate discussions about mental health symptoms and progress [22]. MBC not only enhances treatment of depression, but also empowers and educates patients on their mental health experiences by giving them an active and practical role in their treatment. Indeed, there is a demonstrated link between MBC implementation and improved therapist-patient relationship in youth mental health treatment [13, 15, 33].

While the benefits of MBC have been well documented, there is little information on actual implementation of MBC among adolescents with depression. Furthermore, how closely clinicians are adhering to the MBC model in psychiatric and primary care settings and what instruments are being used is unknown. Finally, there is not a consistent definition of MBC, suggesting the possibility of a wide variety of MBC tools and procedures across the mental healthcare field. The aim of this paper is to describe MBC implementation and fidelity across a variety of clinical settings in the state of Texas.

## Methods

### Study Design and Participant Enrollment into Texas Youth Depression and Suicide Research Network

The Texas Youth Depression and Suicide Research Network (TX-YDSRN) is a collaborative state-wide healthcare learning system established to better understand depression and suicidality among Texas youth. The Network, which was funded by the Texas State Legislature as part of the Texas Child Mental Health Care Consortium, is comprised of 12 academic medical centers that serve as primary research sites (nodes) responsible for recruitment, retention, and data collection for an adolescent depression and suicide registry study. Each node includes and/or partners with three or more clinics (node-subsites) that provide mental health services to youth in primary care, specialty care, inpatient, and outpatient psychiatric treatment settings. Subsites support the Network through participant recruitment and engagement with measurement-based care within their clinics.

Youth are eligible to participate if they are between the ages of 8–20 years, screen positive for depression, suicidality, or are receiving care for depression at one of the participating clinics. Depression is screened via the 2-item Patient Health Questionnaire (PHQ-2; [30] or the 9-item Patient Health Questionnaire for adolescents (PHQ-A, [26], suicide is screened with the Concise Health Risk Tracking – Self Report scale (CHRT-SR; [44, 49] or item 9 of the PHQ-A. The recruiting clinic identifies youth who are engaging in care and connects them to the research study team at their affiliated node, who invites the youth to participate. Written informed consent is obtained from the parent/guardian for youth under 18 years of age along with youth assent, while youth aged 18 years and older provide written informed consent to participate.

An initial screening visit takes place to determine eligibility. If PHQ scores are collected by clinics within seven days of consent as part of routine care, they are utilized to determine eligibility. If no score is available, the research team will complete an initial screening to determine eligibility. Enrolled participants complete a baseline visit in which a series of self-report and assessor administered measures are collected. Follow up visits that include self-report surveys begin at month 1 after baseline and continue bi-monthly for month 2 through month 24. Additional measures administered by assessors occur during visits at months 6, 12, 18, and 24. In alignment with MBC guidelines, all research visits include assessments for depression, anxiety, side effects, and patient adherence (measures used are detailed below). A full description of the registry study is provided in Trivedi, et al. [47].

Clinical care was provided within each of the subsites and was independent of the research registry study. However, a component of the TX-YDSRN initiative was to evaluate the participating healthcare systems’ systematic screening and monitoring of youth depression, with the goal to improve systems of care in the state. As such, the project sought to identify the current state of MBC use and to enhance the utilization of MBC in all participating clinics. A multi-pronged approach was used to characterize MBC across participating clinics and support MBC implementation across the Network.

### Characterizing MBC in Node Subsites

The purpose of the MBC initiative for the TX-YDSRN project is to support the studies’ use and development of the Learning Healthcare System model and to utilize research data to improve clinical care. Prior to site initiation, information was collected about participating clinics, including the model of care provided (i.e., outpatient, inpatient, community mental health, intensive outpatient, day treatment, other), current suicide and depression screening practices, and whether they had a standardized approach to MBC.

### MBC Fidelity Form

To assist with guiding the direction of the MBC initiative, the Network established the MBC Committee, comprised of Network members who were interested in disseminating MBC guidelines. The MBC Committee developed a data collection form to assess MBC fidelity across subsites. The MBC Fidelity form was designed to follow participants throughout their care at the node-subsites for the duration of their enrollment in the Network registry study. The MBC Fidelity form collects details regarding whether and how the patient was assessed for depression and anxiety, side effects, and treatment adherence. Specifically, questions ask if the patient was assessed for these things at the corresponding visit (yes/no), if a standardized instrument was used (as well as which measure and the corresponding score), or if the assessment was based on clinician interview only (yes/no).

Each node-subsite was given the option to provide measurement-based care as part of the clinical workflow to participants enrolled in this study. Research coordinators conducted chart reviews within the Electronic Health Record (EHR) for each clinical visit that occurred after consent to assess MBC fidelity. For some node-subsites where treatment involved daily visits for prolonged periods of time, the MBC Fidelity form was completed weekly rather than daily. The MBC form was revised in May of 2021 to capture more detailed information on measures being used. Data from MBC Form V1.0 (118 records for 64 unique participants) and MBC Form V2.0 (3468 records for 709 unique participants) were combined into one analytical dataset. Duplicate forms filled out for both V1.0 and V2.0 were manually reviewed and the V1.0 was removed if it was identified to be a true duplicate entry. As such, data not captured in V1.0 contributed to missing values in the analyzed data set. Due to the timing of data collection, some clinical visits that occurred prior to May 2021 may be included in V2.0. For purposes of these analyses, we report on the MBC Fidelity at the first clinical visit after consent, as well as the most recent visit for participants with more than one visit.

Descriptive analyses were conducted using SAS and were summarized as frequency and percentages for categorical variables. Continuous variables were summarized as mean ± standard deviation.

## Results

### Subsite Characteristics at Initiation

A total of 73 clinical sub-sites were onboarded as part of the TX-YDSRN across the 12 nodes. Most clinics (78.08%, *n* = 57) were part of one of the 12 large academic medical centers, while roughly a quarter of sites (21.92%, *n* = 16) were community-based. Most clinics engaged with the project included outpatient psychiatry (31.55%), primary care settings (28.77%), and community mental health settings (15.07%). Day treatment programs, inpatient psychiatry, intensive outpatient programs, student health programs, and other types of non-psychiatric specialty care settings were represented across the other clinics (24.61%).

At the time of study initiation, 95.89% (*n* = 70) of sites indicated using a rating scale to assess depression symptoms: 87.67% (*n* = 64) indicated they were using the PHQ-9 to assess for depression, 8.22% (*n* = 6) were using another form of standardized questionnaire, and 4.11% (*n* = 3) stated they were not systematically assessing depression. Participating clinics indicated most screenings for suicidality were from the PHQ-9 questionnaire (82.19%, *n* = 60), 10.96% (*n* = 8) utilized another form of questionnaire to assess for suicidality, while 6.85% (*n* = 5) indicated they conducted no systematic suicide screening.

### MBC Fidelity

Eighteen clinics (two academic sites and all community sites) did not allow access to the EHR; thus, MBC data are only available for 55 sites. MBC Fidelity forms analyzed were collected between 9/18/2020 and 3/23/2023 for a total of 719 unique participants.

#### Depression

Of 719 documented visits collected on the first visit, 92.07% (*n* = 664) of forms indicated depression was assessed; 421 (58.55%) utilized standardized depression rating scales to assess symptoms. Of those using standardized scales, most (*n* = 394, 54.80%) used a version of the PHQ. Of the remaining 242 participants with indicated depression assessment, most used informal non-standardized measures such as Clinical Global Impression Scale [21], mental status exam, informal scale of 1–10, or clinical interview only (*n* = 235, 32.68%). See “First Visit” column in Table 1 for additional detail.

**Table 1:** Depression assessment data from the MBC Fidelity form

Variable	First visit (n = 719)		Last visit (n = 563)	
	n	%	n	%
How was depression assessed?				
Standardized measure				
PHQ-2	9	1.25	5	0.89
PHQ-9	394	54.80	305	54.17
QIDS-16	2	0.28	3	0.53
QIDS-CR	16	2.23	6	1.07
RCADS	0	0	1	0.18
Clinical interview or clinician rating				
CGI	1	0.14	0	0
Mental Status Exam	3	0.42	1	0.18
Informal “out of 10”	2	0.28	2	0.36
Non-specified interview	229	31.85	184	32.68
Other	2	0.28	1	0.18
Not assessed	54	7.51	50	8.88
Missing	7	0.98	4	0.71

PHQ-9 = 9-item Patient Health Questionnaire; PHQ-2 = 2-item Patient Health Questionnaire; QIDS-16 = 16-item self-report Quick Inventory of Depressive Symptomatology; QIDS-CR = clinician rated Quick Inventory of Depressive Symptomatology; RCADS = Revised Children’s Anxiety and Depression Scale; CGI = Clinical Global Impression

For those with multiple forms (*n* = 563), the most recent form collected was assessed and indicated 90.94% (*n* = 512) assessed for depression; 320 (56.84%) utilized a standardized depression rating scale with a version of the PHQ being used most frequently (*n* = 310, 55.06%). Almost all remaining forms indicated the use of informal non-standardized measures (*n* = 187, 33.21%)*.* See “Last Visit” column in Table 1 for additional detail.

#### Anxiety

Of 719 documented visits collected on the first form, 78.86% (*n* = 567) of forms indicated anxiety was assessed. Of these, 243 (42.86%) utilized standardized anxiety rating scales to assess symptoms. Of those using standardized scales, almost all used (42.33%, *n* = 240) a version of the Generalized Anxiety Disorder – 7 item (GAD-7) [41], with a select few (*n* = 3) using the Screen for Child Anxiety Related Disorders (SCARED) [7]. The remaining forms indicated the use of informal non-standardized measures for anxiety assessment (e.g., Clinical Global Impression Scale, mental status exam, verbal questions, and informal scale of 1–10) or clinical interview only (44.65%, n = 321). See “First Visit” column in Table 2 for full breakdown.

**Table 2:** Anxiety assessment data from the MBC Fidelity form

Variable	First visit (n = 719)		Last visit (n = 563)	
	n	%	n	%
How was anxiety assessed?				
Standardized measure				
GAD-2	68	9.46	53	9.41
GAD-7	172	23.92	145	25.75
SCARED	3	0.42	1	0.18
RCADS	0	0	1	0.18
Clinical interview or clinician rating				
CGI	6	0.83	13	2.31
Mental Status Exam	1	0.14	0	0
Informal “out of 10”	5	0.70	6	1.07
Non-specified interview	308	42.84	234	41.56
Other	1	0.14	1	0.18
Not assessed	151	21.00	106	18.83
Missing	4	0.53	3	0.53

GAD-7 = 7-item Generalized Anxiety Disorder questionnaire; GAD-2 = 2-item Generalized Anxiety Disorder questionnaire; SCARED = Screen for Child Anxiety and Related Emotional Disorders; RCADS = Revised Children’s Anxiety and Depression Scale; CGI = Clinical Global Impression

For those with multiple forms (*n* = 563), the most recent form collected indicated 80.64% (*n* = 454) assessed for anxiety, with 200 (35.52%) utilizing a standardized anxiety rating scale (GAD-7 most frequently [*n* = 198, 35.17%]), with less than 1.00% (*n* = 2) using the SCARED and Revised Children’s Anxiety and Depression Scale (RCADS) [11]. All remaining forms indicated the use of informal measures or clinical interview only (*n* = 254, 45.12%)*.* See “Last Visit” column in Table 2 for full breakdown.

#### Side Effects

Forms collected at the first documented visit indicated assessment of side effects at 71.79% (*n* = 509) of visits. The Frequency, Intensity, & Burden of Side Effects Rating – Child [42] was the only standardized instrument used to assess for side effects (4.59%, *n* = 33) at the first documented visit. No other standardized instrument was used to assess side effects with 66.20% (*n* = 476) indicating side effects were assessed based on clinician interview only. See “First Visit” column in Table 3 for additional detail.

**Table 3:** Side effects assessment data from the MBC Fidelity form

Variable	First visit (n = 719)		Last visit (n = 563)	
	n	%	n	%
How were side effects assessed?				
Standardized measure				
FIBSER-C	33	4.59	47	8.35
Clinical interview or clinician rating				
Non-specified interview	476	66.20	372	66.07
Not assessed	199	27.68	133	23.62
Missing	11	1.53	11	1.95

FIBSER-C clinician rated Frequency, Intensity, and Burden of Side Effects Rating scale

For those with multiple forms, an assessment of side effects occurred 74.42% of the time based on the most recent visit assessed (*n* = 419). The FIBSER-C was the only standardized instrument used to assess for side effects at the most recent documented visit (8.34%, *n* = 47). No other standardized instrument was used to assess side effects with 66.07% (*n* = 372) indicating side effects were assessed based on clinician interview only. See “Last Visit” column in Table 3 for additional detail.

#### Adherence

Based on forms collected at the first documented visit, 70.79% (*n* = 509) indicated treatment adherence was assessed during the visit. Only 4.73% of those assessing for treatment adherence utilized the Patient Adherence Questionnaire–Revised (PAQ-R) [53] (*n* = 34). Of the remaining forms that assessed for treatment adherence, none indicated a separate standardized instrument was used; all (*n* = 471) indicated the assessment was based on clinician interview only. See “First Visit” column in Table 4 for additional detail.

**Table 4:** Medication adherence assessment data from the MBC Fidelity form

Variable	First visit (n = 719)		Last visit (n = 563)	
	n	%	n	%
How was medication adherence assessed?				
Standardized measure				
PAQ	34	4.73	49	8.70
Clinical interview or clinician rating				
Non-specified interview	471	66.51	389	69.09
Not Assessed	200	27.82	118	20.96
Missing	14	1.95	7	1.24

PAQ = Patient Adherence Questionnaire

For forms collected across multiple visits, 78.73% (*n* = 441) indicated treatment adherence was assessed during the most recent visit. Slightly more than the first visit assessed, 8.70% of those assessing for treatment adherence utilized the PAQ-R (*n* = 49). Of the remaining forms showing assessment of treatment adherence, none indicated a separate standardized instrument was used. Nearly all (*n* = 389) indicated the assessment was based on clinician interview only. See “Last Visit” column in Table 4 for additional detail.

#### Medication Changes and Follow-up Appointments

Forms collected at the first documented visit indicated 43% (*n* = 311) of patients had medication changes at the visit. For most participants (80%), there was a recommendation that a follow-up appointment be scheduled. A review of forms collected across multiple visits indicated 35% (*n* = 199) had a medication change during the most recent visit with their clinician and 73% were recommended a follow-up visit.

## Discussion

The use of MBC to address youth depression is urgently needed to improve identification, assessment, and treatment for this serious illness. MBC is commonly used in other applied health disciplines, yet, despite mounting evidence of its benefits and the manageable barriers to its implementation, its adoption within psychiatric practice has lagged. Herein, we presented a model for measuring and reviewing MBC practices that was utilized across several sites in Texas.

At study initiation, the partnering clinics reported they were using measures to consistently screen for depression, with 95.89% reporting use of some standardized measure of depression and 93.15% reporting using some measure with a suicide risk item. It is unclear the frequency with which these measures were being utilized based on the site initiation data. The PHQ-9 was the most utilized depression screener, with 87.67% of clinics reporting use; this is not surprising given the PHQ-9 and its variations (e.g., PHQ-2, PHQ-A) have been well validated for pediatric use and are widely used in the research literature [19]. However, most clinics reported obtaining data on suicide risk using an item included within a depression screener. The use of a specific suicide risk screening tool, such as the Ask Suicide-Screening Questions (ASQ), is recommended, as depression screens under-detect suicidal ideation [8, 24].

Upon review of MBC fidelity, assessment of depression was confirmed, with over 90% indicating some level of assessment of depression within the EHR; however, far fewer were using a standardized depression rating scale; in fact, of the full sample, only 59% were using a standardized depression rating scale. More than a third of visits indicated clinical interview as the only assessment being used, despite over 95% had previously reported using a standard measure. Use of just a clinical interview does not align with MBC and indicates limited fidelity to the MBC model. These rates remain consistent when the most recent visit data is assessed when multiple forms are available for review. This suggests that those using a standardized measure are doing so consistently, which is representative of an MBC model. Fewer clinics formally assessed for anxiety using a standardized measure, with the GAD-7 being the most frequently used. Given the high comorbidity of depression and anxiety in youth [16] and the relationship between baseline anxiety and poorer treatment response in depressed youth [54], data about anxiety symptoms in youth being treated for depression is needed [51]. Far fewer clinics utilized standardized measures of side effects and adherence, in fact, less than 5% of visits indicated that a systematic rating scale was used to assess side effects or adherence to treatment. The MBC Fidelity form did not collect information on what medications were currently prescribed, so it is possible that side effects and adherence were not applicable if participants were not currently taking medication. Despite limited use of measures for side effects and adherence, medication changes occurred in almost half of the visits. Given the role of medication side effects in treatment decisions and youth and family adherence [43], it is important for this to be systematically assessed as part of the MBC process.

One important finding during the MBC initiative was that each node and subsite operated under a different definition of MBC. While subsites reported that they used MBC prior to site initiation, it was clear that assessing MBC fidelity was key to get a more accurate sense of the state of care. Thus, the MBC Fidelity form was developed to capture all potential aspects of MBC being utilized. These results clearly indicate that while many clinicians believed they were adequately assessing depression and anxiety symptoms, only about 60% were using ratings scales to systematically assess symptoms, whether at the first visit or later in follow-up care. Possibly more striking is that 30% of visits did not have any documentation that side effects or adherence to treatment was assessed at all; only 6% assessed side effects or adherence with a rating scale. Thus, formalizing a standard definition of MBC and training providers about available rating scales is an important next step.

### Limitations

There were several limitations of the MBC initiative of the TX-YDSRN. First, completion of the MBC Fidelity form was based on the perception of the research coordinator collecting the data via chart review through node-subsite EHR systems. Furthermore, not all node-subsites use the same EHR thereby limiting the standardization of data collection. Thus, it is possible that some clinicians or sites did obtain rating scales that were not entered into the EHR. However, based on the data from the fidelity ratings, the percentages of rating scales used was relatively similar to the pre-initiation reports. Second, not all sites gave access to their EHR, which presents a gap in data collection. Engaging with sites to gain access to their EHR could improve the representation of various settings when assessing MBC. Node leadership continues to work with these clinics to broker access. Third, the Hub, who is responsible for data quality, does not have the ability to cross check MBC Fidelity forms with EHRs to ensure a form was completed for every clinical visit a participant may have attended. In addition, these analyses only examined the first and most recent visit, so it is possible that interim visits may have had differing reports. Fourth, as data collection was limited to TX-YDSRN participants only, we are unable to assess how the characteristics of other clinic patients (age, primary language, disorder type, etc.) may have affected the clinics standard operating procedures and decisions regarding the use of measurements.

**Fig. 1:**
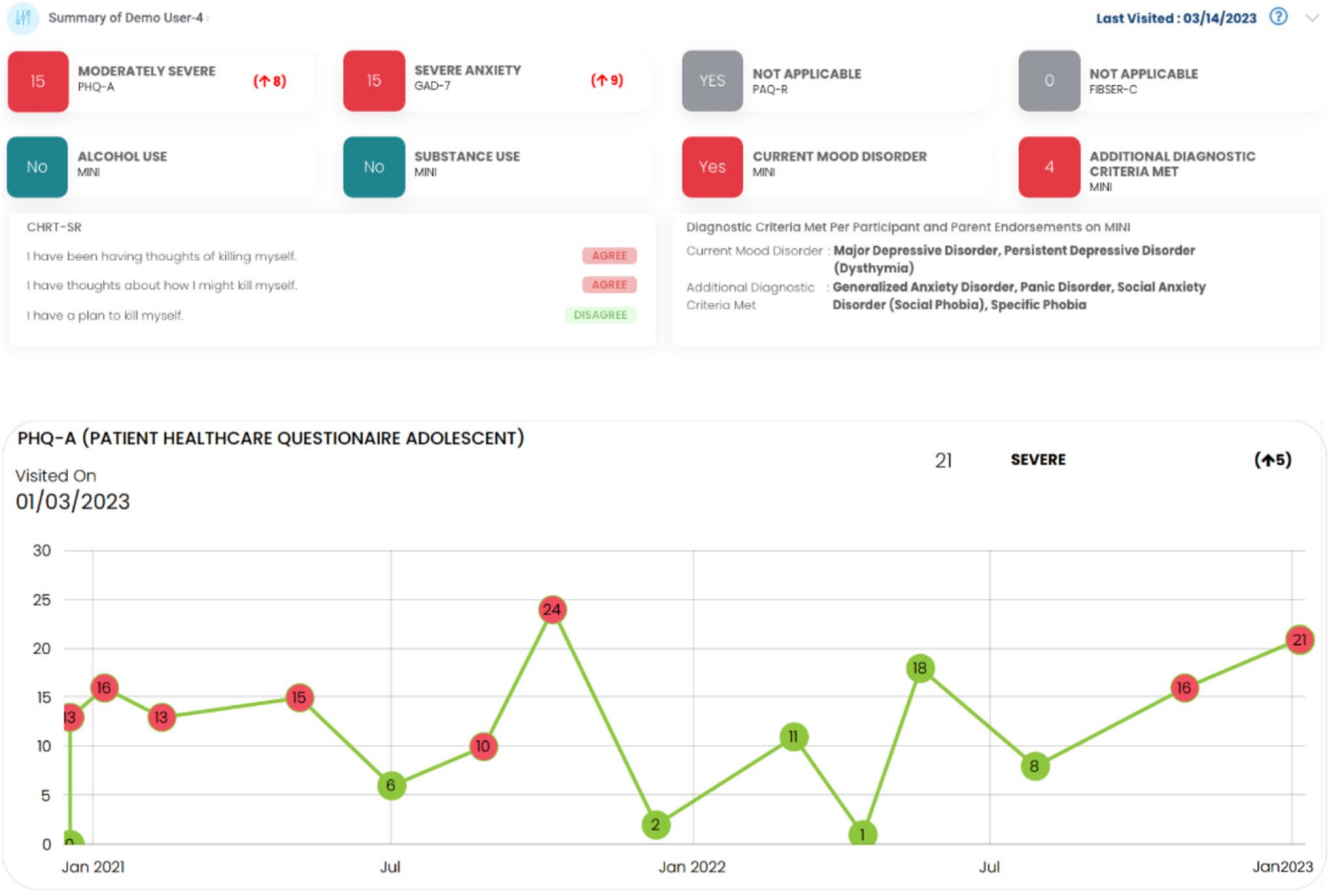
**A** Appearance of the clinical dashboard summary. **B** Longitudinal patient research data reported to the node subsite clinicians

Finally, MBC guidelines recommend treatment decisions be based on real time feedback from the standardized clinical measures. Many of the subsites reported using electronic versions of measures, yet it is unclear if the clinicians actually reviewed those measures during the visit. To support MBC efforts across the diverse settings represented in TX-YDSRN, a clinical dashboard was created to provide real-time feedback to treating clinicians on measures being collected as part of the registry study (albeit at the research visit, not necessarily the clinical visit). Participants were allowed to opt out of sharing research data with their treating clinician. However, for those youth and parents who consented to share data, these interactive dashboards include a summary of the most recent research scores collected for depression (PHQ-A), suicidality (CHRT-SR and Concise Associated Symptom Tracking—Self Report [CAST-SR]; [44, 49], anxiety (GAD-7), medication adherence (PAQ-R), and side effects (FIBSER-C). Current scores that indicate an increase in symptomology, non-medication adherence, and unacceptable side effects are color coded to draw attention to areas to be explored further by the clinician [see Fig. 1A]. Furthermore, longitudinal data for each scale is provided for the clinician to review all data points collected across multiple research visits to better inform care [see Fig. 1B].

The dashboard was designed to be user friendly and incorporate key clinical information in an easy-to-use format. Feedback from clinicians across the network was incorporated prior to the launch of the dashboards with additional revisions and features being added to enhance the usability and encourage utilization across different settings. The dashboards are part of a separate system that is not integrated into the existing EHRs across sites. This requires clinicians to request access to the dashboard and create a unique login to access participant research data. To mitigate this barrier, a report was developed to summarize research data from the dashboard for individual participants who consented to sharing data with their clinical provider(s). Research coordinators with access to the EHR download the summary report, upload a copy into the participant’s health record, and notify the corresponding clinician of the updated data. Anecdotal feedback from clinicians indicates this is most useful when updated immediately before a patient has a scheduled visit. Some of the data collected in the MBC Fidelity form may be reflective of the research data being provided to clinicians in the EHR rather than formal assessments built into their existing workflows.

## Recommendation for Next Steps

Implementation models such as those used in quality improvement projects in other facilities should be considered to support the adherence to MBC practices in clinical settings. Such models consider individual clinic workflows and yield specific recommendations for when measurements should be collected. Future efforts to better aid providers in automatically pulling data to assess screening rates and fidelity to MBC practices, such as administering measurements upon follow up visits and adjusting treatment based on outcomes, are needed. Tools are available to assist providers, clinics, and systems in implementing and improving the quality of MBC. For example, the VitalSign6 program, a point-of-care, Web-based application, has been successfully used in pediatric primary and specialty care settings [36, 46].

To increase engagement with MBC, different platforms should be utilized to increase access to MBC trainings. Online self-paced modules that include case studies and quizzes, in addition to live sessions, may be beneficial to provide more flexibility and access to training material. Suggestions such as providing continuing education credits for attending trainings should be considered. More on-site support from the MBC Committee and Hub may increase the baseline level of MBC engagement and interest for sub-sites joining the study as well as to provide the ability to collaborate on ways in which to increase fidelity to MBC.

## Summary

Utilization of MBC has demonstrated positive outcomes for patients with depression; however, widescale implementation in clinical settings still has not become the norm. Without a mechanism to assess for fidelity it is difficult to determine how well clinics adhere to an MBC model. A primary goal for this project was to characterize MBC fidelity across the state and describe what measures, if any, the clinics participating in TX-YDSRN used. The MBC Fidelity form was developed to gather MBC information via chart review. While nearly all sites indicated they used a standardized measure for depression at study initiation, in practice only 59% of first documented visits indicated use of a standardized measure. Even fewer (43%) indicated assessment of anxiety with a standardized measure. Side effects and medication adherence, a key component of MBC, were assessed for roughly 75% of first documented visits, but less than 5% used a standardized measure. Similar findings were seen at the most recent visit documented. This disconnect suggests that while most clinicians believe they are using MBC, they may not adhere fully to an MBC model. Developing a standard definition of MBC and providing additional training to providers will assist with broader implementation of MBC, and ensure it is done with absolute fidelity.

## Data Availability

TX-YDSRN data is not publicly available.
